# The Role of Oral Microbiota in Intra-Oral Halitosis

**DOI:** 10.3390/jcm9082484

**Published:** 2020-08-02

**Authors:** Katarzyna Hampelska, Marcelina Maria Jaworska, Zuzanna Łucja Babalska, Tomasz M. Karpiński

**Affiliations:** 1 Department of Genetics and Pharmaceutical Microbiology, Poznań University of Medical Sciences, Święcickiego 4, 60-781 Poznań, Poland; kasia-hampelska@wp.pl (K.H.); rufin1985@interia.pl (M.M.J.); 2 Central Microbiology Laboratory, H. Święcicki Clinical Hospital, Poznań University of Medical Sciences, Przybyszewskiego 49, 60-355 Poznań, Poland; 3 Chair and Department of Medical Microbiology, Poznań University of Medical Sciences, Wieniawskiego 3, 61-712 Poznań, Poland; z.babalska@gmail.com

**Keywords:** halitosis, malodor, volatile sulfur compounds, hydrogen sulfide, microbiota, *Fusobacterium*, *Porphyromonas*, *Prevotella*, periodontitis, carcinogenesis

## Abstract

Halitosis is a common ailment concerning 15% to 60% of the human population. Halitosis can be divided into extra-oral halitosis (EOH) and intra-oral halitosis (IOH). The IOH is formed by volatile compounds, which are produced mainly by anaerobic bacteria. To these odorous substances belong volatile sulfur compounds (VSCs), aromatic compounds, amines, short-chain fatty or organic acids, alcohols, aliphatic compounds, aldehydes, and ketones. The most important VSCs are hydrogen sulfide, dimethyl sulfide, dimethyl disulfide, and methyl mercaptan. VSCs can be toxic for human cells even at low concentrations. The oral bacteria most related to halitosis are *Actinomyces* spp., *Bacteroides* spp., *Dialister* spp., *Eubacterium* spp., *Fusobacterium* spp., *Leptotrichia* spp., *Peptostreptococcus* spp., *Porphyromonas* spp., *Prevotella* spp., *Selenomonas* spp., *Solobacterium* spp., *Tannerella forsythia*, and *Veillonella* spp. Most bacteria that cause halitosis are responsible for periodontitis, but they can also affect the development of oral and digestive tract cancers. Malodorous agents responsible for carcinogenesis are hydrogen sulfide and acetaldehyde.

## 1. Introduction

Halitosis is a common problem that manifests as an unpleasant and disgusting odor emanating from the mouth [1]. Malodor is mainly caused by putrefactive actions of microorganisms on endogenous or exogenous proteins and peptides. Oral malodor is an embarrassing condition that affects a large percentage of the human population. This condition often results in nervousness, humiliation, and social difficulties, such as the inability to approach people and speak to them [2,3,4,5,6]. Halitosis experiences from about 15% to 60% of the human population worldwide [7,8,9,10,11,12]. Halitosis can be divided into extra-oral halitosis (EOH) and intra-oral halitosis (IOH) [2,3,5]. 

The factors that increase the likelihood of halitosis include periodontal diseases, dry mouth, smoking, alcohol consumption, dietary habits, diabetes, and obesity. Halitosis can also be affected by the general hygiene of the body (i.e., dehydration, starvation, and high physical exertion), advanced age, bleeding gums, decreased brushing frequency, but also by stress [3,13,14,15,16]. Produced during stress, catecholamines and cortisol increased hydrogen sulfide production by sub-gingival anaerobic bacteria [17]. The medications which can cause extra-oral halitosis were categorized into 10 groups: acid reducers, aminothiols, anticholinergics, antidepressants, antifungals, antihistamines and steroids, antispasmodics, chemotherapeutic agents, dietary supplements, and organosulfur substances [18].

More and more patients are struggling with bad breath and report this problem to their primary care practitioner for diagnosis and management [19,20]. However, many physicians, dentists, and biologists have insufficient knowledge regarding the cause and biochemistry of this disease. 

In this review, we focused on intra-oral halitosis, regardless of classification.

## 2. Classifications of Halitosis

**Figure 1:**
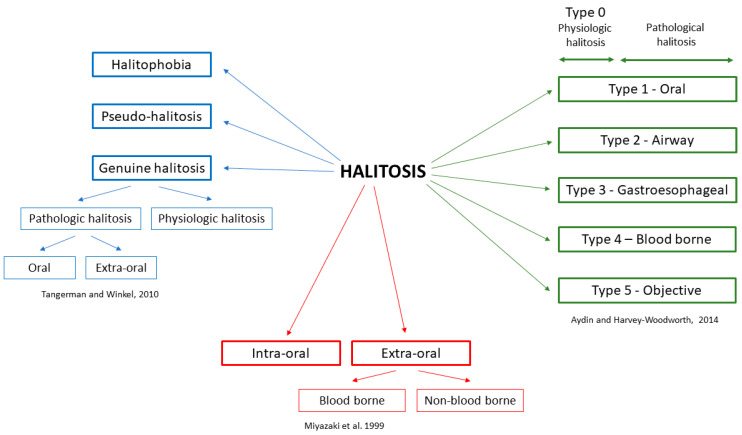
Classifications of halitosis [21,22,23,24].

In the literature, mainly three classifications of halitosis are used, described by Miyazaki et al., 1999 [21], Tangerman and Winkel in 2010 [22], and Aydin and Harvey-Woodworth in 2014 [23] (Figure 1). 

Miyazaki et al. divided halitosis as intra-oral (IOH) and extra-oral (EOH) [21]. Extra-oral halitosis can be of bloodborne or non-bloodborne origin and covers about 5–10% of all halitosis [22]. Bloodborne-related causes include diabetes metabolic disorders, kidney and liver diseases, and certain drugs and food. Non-bloodborne-related causes include respiratory and gastrointestinal diseases. Meanwhile, pathological conditions in the oral cavity are responsible for 80–90% of IOH [2,3,25]. Both aerobic and anaerobic bacteria can be responsible for IOH. These microorganisms tend to produce foul-smelling, sulfur-containing gases called volatile sulfur compounds (VSCs) [23,26].

In the classification of Tangerman and Winkel [22], halitosis is classified as genuine and delusional. Delusional halitosis (monosymptomatic hypochondriasis; imaginary halitosis) is a condition in which patients believe that their breath is smelly and offensive. The social pressure of having fresh smelling breath increases the number of people that are preoccupied with this condition. However, the perception of oral malodor does not always reflect actual clinical oral malodor [27]. Self-perceived halitosis was found to be more prevalent amongst males, particularly smokers, compared to females. However, there are no statistical differences when comparing with different age groups [28]. Genuine halitosis is further subdivided into physiological and pathological halitosis. Physiological halitosis (foul morning breath, morning halitosis) is caused by saliva retention, as well as the putrefaction of entrapped food particles. Meanwhile, intra- and extra-oral causes are responsible for pathological halitosis [3,4,19].

Aydin and Harvey-Woodworth divided pathologic halitosis into five types: Type 1 (oral), Type 2 (airway), Type 3 (gastroesophageal), Type 4 (blood-borne) and Type 5 (subjective). Moreover, it is Type 0 halitosis (physiologic odor), which can be a connection of the physiologic contributions of oral, airway, gastroesophageal, blood-borne, and subjective halitosis. Any combination of the above types can be present in every healthy person [23].

## 3. Volatile Compounds

Halitosis is formed by volatile compounds, which are produced mainly by bacteria in the oral cavity. In the oral cavity, nearly 700 different compounds have been detected [29]. To these volatile substances belong sulfur compounds, aromatic compounds, amines, short-chain fatty or organic acids, alcohols, aliphatic compounds, aldehydes, and ketones (Table 1) [25,30,31,32,33]. It is considered that hydrogen sulfide, methyl mercaptan, and dimethyl sulfide are the main volatile compounds in IOH [34,35,36,37]. In many studies, the measurement of malodor substances concerns only volatile sulfur compounds (VSCs). The most commonly used are VSC monitors, such as the Halimeter (Interscan, Chatsworth, USA) [11,36,38,39,40,41]. This method has a significant disadvantage because the measure of dimethyl sulfide is not exact [42]. Moreover, the presence of alcohols, phenyl compounds, and polyamines can interfere with readings [16,43]. For this reason, in the assessment of IOH, other substances are often not taken into account. However, they can have an equally important role. It is confirmed by studies using gas chromatography-mass spectrometry [29,32,44]. In the paper of Monedeiro et al., in the persons with IOH, 85 volatiles, were detected, and the most predominant classes of malodor compounds were alcohols and ketones. In this group, in comparison to healthy persons, an increased number of volatile sulfur compounds and esters was observed. Simultaneously, authors found ten VSCs substances: methyl thioacetate, dimethyl disulfide, dimethyl trisulfide, dimethyl tetrasulfide, dimethyl pentasulfide, dimethyl sulfone, allyl thiocyanate, allyl isothiocyanate, S-methyl pentanethioate, and thiolan-2-one [44]. In other studies, in halitosis patients, the 30 most abundant volatile compounds in the oral cavity belonged to alkanes or alkane derivatives, therein methyl benzene, tetramethyl butane, and ethanol [45]. Dadamio et al. reported VSC and amines (such as putrescine, cadaverine, and trimethylamine) as the most abundant organic compounds in IOH patients [46].

In Table 1, among others, values of odor thresholds are presented. Amid VSCs, which are the most often described compounds in IOH, the lowest value of odor threshold has methyl mercaptan, followed by hydrogen sulfide and dimethyl sulfide. This means that these substances are mainly responsible for the unpleasant smell in the mouth. Besides, methyl mercaptan is felt in much lower concentrations than the other compounds.

In the oral cavity, the most relevant anatomical part related to IOH is the tongue. The tongue-associated microbiota produce malodorous compounds and fatty acids. The VSCs are the most essential substances responsible for malodor. They are products of metabolism of sulfur amino acids: methionine, cysteine, and homocysteine in the Gram-negative anaerobic bacteria [25,30,47,60]. Hydrogen sulfide and mercaptans are the principal end products [38]. In healthy volunteers, the concentration of H_2_S in saliva was within a range of 1.641–7.124 μM [61]. In other studies, the mean amount of H_2_S in the saliva of healthy persons was 0.5 ng/10 mL, whereas in patients with IOH it was 6.7 ng/10 mL [62]. Gram-positive bacteria can support Gram-negative anaerobic bacteria in the production of VSC. They cut off sugar chains from glycoproteins and provide proteins that are necessary for proteolytic processes [60]. *Streptococcus salivarius* has an impact on the deglycosylation of salivary glycoproteins, mainly mucins, which can next be degraded to VSC by *Porphyromonas gingivalis* [63]. In turn, *Solobacterium moorei* is associated with the production of VSC through β-galactosidase activity and the degradation of glycoproteins [60,64].

The essential VSCs are hydrogen sulfide, dimethyl sulfide, dimethyl disulfide, and methyl mercaptan [25,30] (Table 1). These are produced mostly by anaerobic bacteria. The increased production of malodorous gases occurs mainly in tongue coating, and diseases such as gingivitis and periodontitis and, to a less extent, in pericoronitis, oral ulcers, periodontal abscesses, and herpetic gingivitis [65]. Other volatile organoleptic compounds, such as indole, skatole, amines, and ammonia, are produced by the putrefaction of non-sulfur containing amino acids (i.e., tryptophan, lysine and ornithine). Studies have shown that volatile sulfur compounds are the major contributors to bad breath. Hydrogen sulfide, methyl mercaptan and, to a lesser extent, dimethyl sulfide, represent 90% of the volatile sulfur compounds in halitosis [2,27].

Volatile sulfur compounds can be toxic for human cells even at low concentrations. They contain thiols (-SH groups) that interact with other proteins and support the negative interaction of bacterial antigens and enzymes. The result of this effect is chronic inflammation, periodontal gingivitis, and periodontitis [66]. In human gingival fibroblasts, H_2_S activates the mitochondrial pathway of apoptosis [67]. The H_2_S is a known genotoxic agent, which has an impact on genomic instability and cumulative mutations [68]. In studies on rats, it was demonstrated that hydrogen sulfide leads to ultrastructural changes in epithelial cells and periodontal destruction [69]. Increased amounts of H_2_S by the activation of proliferation, migration, and invasion can also lead to carcinogenesis [70,71]. *Fusobacterium nucleatum* and *Porphyromonas gingivalis* belong to the most essential carcinogenic oral bacteria producing VSCs [70,72]. Cancerogenic is also acetaldehyde produced from ethanol by mucosal epithelial cells or oral microflora, e.g., *Candida albicans*, *Candida* non-*albicans*, *Neisseria* sp., and *Streptococcus* sp. Acetaldehyde binds to DNA and leads to the formation of DNA adducts, point mutations, and DNA cross-linking [73,74]. 

**Figure 2:**
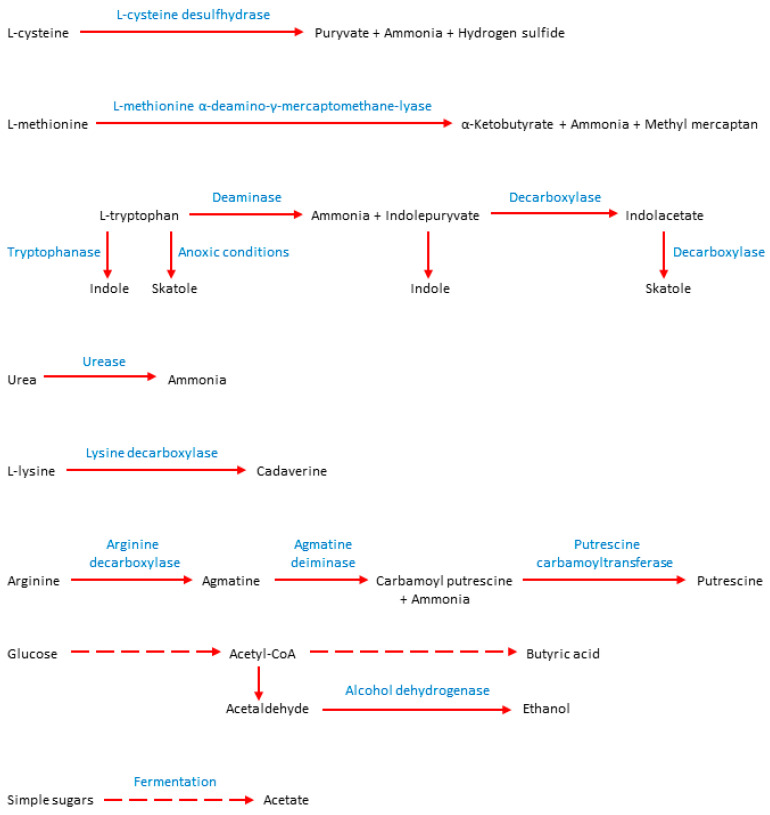
Simplified ways of bacterial production of selected odorous compounds [30,75,76,96,97,98,99,100,101,102].

Other important substances causing IOH are diamines, such as putrescine and cadaverine. Both compounds are produced from amino acids, putrescine from arginine, and cadaverine from L-lysine [75,76] (Figure 2). Both diamines are associated with the putrefaction of food by bacteria occurring in the dental plaque and severe periodontitis [77]. 

Gram-negative bacteria, mostly Enterobacteriaceae, which can colonize the oral cavity and dentures, produce urease that hydrolyzes urea into carbon dioxide and ammonia [78]. *Escherichia coli* can form ammonia from cysteine using cysteine desulfhydrase [79] or reduce nitrates to ammonia [73]. Major contributors to trimethylamine production are gut bacteria, which can be inhabitants of the oral cavity, such genera as *Anaerococcus*, *Clostridium*, *Collinsella*, *Desulfovibrio*, *Lactobacillus*, *E. coli*, *Citrobacter*, *Edwardsiella*, *Providencia*, and *Proteus* [74,80,81,82,83,84].

Indole and skatole are produced in high amounts by intra-oral, Gram-positive *Streptococcus milleri*, and anaerobic Gram-negative bacteria such as *Porphyromonas intermedia*, *Fusobacterium nucleatum*, and *Porphyromonas gingivalis*. Small amounts of both aromatic compounds produced *Aggregatibacter aphrophilus*, *Staphylococcus epidermidis*, and *Streptococcus sanguis* [85].

## 4. Microbiota Responsible for Intra-Oral Halitosis

The human oral cavity microbiota is an ecosystem consisting of various symbiotic microbes. There is a relationship between the global composition of indigenous bacterial populations and human health [86,87]. The oral microbiota is truly diverse and consists of 50–100 billion bacteria. There are about 700 taxa, of which one-third cannot be grown in vitro [88,89]. A vast range of microorganisms inhabit the human oral cavity, including bacteria, fungi, viruses, and protozoa [90,91]. The basic oral microbiota consists of phyla, such as Firmicutes, Proteobacteria, Fusobacteria, Bacteroidetes, and Actinobacteria. The most dominant genera are *Streptococcus*, *Veillonella*, *Gemella*, *Granulicatella*, *Neisseria*, *Haemophilus*, *Selenomonas*, *Fusobacterium*, *Leptotrichia*, *Prevotella*, *Porphyromonas*, and *Lachnoanaerobaculum*. Lots of current findings reported that oral bacteria can be biomarkers that differentiate healthy and pathological conditions within the oral cavity. The oral microbiota research is used as a diagnostic and prognostic tool in the aspect of human health. In the human body, the oral cavity is the second site, after the colon, containing the largest diversity of microbial populations [92]. Simultaneously, changes in the gut microbiota are reflected in the oral microbiota, and the microbial communities of the oral cavity and gastrointestinal tract are predictive of each other [93,94,95]. 

The oral bacteria that are most likely to produce hydrogen sulfide from L-cysteine or serum are *Bacteroides* spp., *Eubacterium* spp., *Fusobacterium* spp., *Peptostreptococcus* spp., *Porphyromonas* spp., *Selenomonas* spp., *Tannerella forsythia*, and *Veillonella* spp. Another essential component of VSC is methyl mercaptan produced from L-methionine or serum. It is a metabolic product mainly derived from *Bacteroides* spp., *Eubacterium* spp., *Fusobacterium* spp., *Porphyromonas* spp., and *Treponema denticola* [30,96] (Table 2).

Ye at al.’s studies showed a correlation between high H_2_S and CH_4_S levels and the growth of microorganisms such as *Prevotella* spp., *Peptostreptococcus* spp., *Eubacterium nodatum*, and *Alloprevotella* spp. Comparing the study and control group, the authors noted significantly higher concentrations of all compounds (total VSC, H_2_S, CH_4_S, and C_2_H_6_S) in the malodor group [103]. The most active producers of hydrogen sulfide are Gram-negative anaerobes *Prophyromonas gingivalis*, *Treponema denticola*, and *Tannerella forsythia* (red complex). Furthermore, the red complex microorganisms are associated with periodontal disease. Hydrogen sulfide and methyl mercaptan are produced in large quantities in periodontal inflammations [104,105,106]. During periodontitis, *Porphyromonas* spp., *Prevotella* spp., and *Treponema denticola* may play the most crucial role in providing amino acids to other anaerobic bacteria. Through this process, anaerobes acquire the opportunity to produce H_2_S and CH_4_S [60] (Figure 2). In the studies of Takeshita et al., the producers of hydrogen sulfide in saliva were bacteria from the genera *Neisseria*, *Fusobacterium*, *Porphyromonas*, and SR1. In contrast, producers of the methyl mercaptan are representatives of the genera *Prevotella*, *Veillonella*, *Atopobium*, *Megasphaera*, and *Selenomonas* [107]. Significant contributors to methyl mercaptan production are also gut bacteria, which can be inhabitants of the oral cavity, such as *E. coli*, *Citrobacter* spp., and *Proteus* spp. [48,84].

Many studies showed that bacterial diversity in the group of patients with IOH is much higher than in the control group. Furthermore, many publications draw attention to the correlation between halitosis and individual microorganisms. The relationship between tongue bacterial composition structure and VSC gases is also mentioned by many authors [3,108]. Many oral bacteria that cause IOH contain similar enzymes. These enzymes are proteins encoded by related genes (*megL*, *lcs*, *mgl*) in the genomes of various bacterial species. The main enzymes are methionine γ-lyase, L-cysteine desulfhydrase, and L-methionine α-deamino-γ-mercaptomethane-lyase [109].

Veloso et al. mentioned that in 85% of the patients IOH is caused by Gram-negative bacteria [6]. According to Wei et al., the oral microbiota responsible for IOH includes a wide range of microbial communities, including 13 phyla, 23 classes, 37 orders, 134 genera, 266 species, and 349 operational taxonomic units. The largest percentage amongst the oral cavity microorganisms are genera, like *Prevotella*, *Alloprevotella*, *Leptotrichia*, *Peptostreptococcus*, and *Stomatobaculum*. These bacteria present a higher percentage of occurrence in the sample of patients with IOH than in the control samples from healthy patients [103]. In turn, the presence of bacteria, such as Firmicutes, Proteobacteria, Bacteroidetes, Actinobacteria, and Fusobacteria, was demonstrated in both the samples from examined and control groups. Firmicutes was the most abundant phylum in saliva samples from both groups [110,111].

The composition of the tongue microbiota has an essential influence on IOH. The most common molecular technique for testing and evaluating an oral cavity microbiome is the sequencing [5,107,112,113]. Seerangaiyan et al. published a review in 2017, in which they showed the composition of the bacteria of *Aggregatibacter*, *Campylobacter*, *Capnocytophaga*, Clostridiales, *Leptotrichia*, *Parvimonas*, *Peptostreptococcus*, *Peptococcus*, *Prevotella*, *Selenomonas*, *Dialister*, *Tannerella*, and *Treponema* in the group of patients with IOH. Using the amplification of 16S rRNA, the researchers also demonstrated a high prevalence of *Solobacterium moorei* strains in the IOH group. By testing the control group, significant differences were found in both groups of healthy and sick people. Furthermore, using polymerase chain reactions (PCRs), Seerangaiyan et al. showed the positive correlation of *Leptotrichia* spp. and *Prevotella* spp. to oral malodor severity, contrary to *Haemophilus* spp., *Gemella* spp. and *Rothia* spp. [5].

Patients with IOH have a specific biofilm on the dorsal part of the tongue. Bernardi et al. stated that this biofilm consists of a significant proportion of *Fusobacterium nucleatum* and *Streptococcus* spp. The occurrence of these two types of bacteria in patients with IOH was completely related. According to the authors, these microorganisms contribute significantly to IOH and can be treated as treatment targets [114]. In other research, Bernardi and partners showed that *Actinomyces graevenitzii* and *Veillonella rogosae* were closely related to the occurrence of IOH in a group of volunteers. Also, *Streptococcus mitis*/*oralis*, *S. pseudopneumoniae*, and *S. infantis*, as well as *Prevotella* spp. were detected often in malodor patients. Moreover, following the earlier findings, the researchers’ results revealed the presence of *Actinomyces odontolyticus*, *Solobacterium moorei*, *Prevotella melaninogenica*, *Fusobacterium periodonticum*, and *Tannerella forsythia* in IOH patients. Furthermore, microorganisms such as *Streptococcus parasanguinis*, *S. salivarius*, *Veillonella* spp., and *Rothia mucilaginosa* dominated in the oral microbiota of healthy people [112].

Yitzhaki et al. noticed the connection between IOH and wearing dentures. The unpleasant odor was organoleptically assessed and the oral microbiome was analyzed using Next Generation Sequencing 16S rDNA technology. Researchers have identified bacterial taxa, including nine phyla, 29 genera, and 117 species. The samples taken from patients with IOH showed the dominance of the phyla Firmicutes and Fusobacteria and the genera *Leptotrichia*, *Atopobium*, *Megasphaera*, *Oribacterium*, and *Campylobacter*. The analyses revealed a significant diversity of the oral microbiota among samples from IOH patients wearing alveolar dentures and significant differences in comparison to the control group [113].

The use of tobacco also has a huge impact on the oral microbiota diversity. After examining a group of smokers and non-smokers, researchers reported that in both groups, most of the oral microbiota were Gram-negative bacterial strains. Simultaneously, *Klebsiella pneumoniae* dominated in smokers’ saliva and *Pseudomonas aeruginosa* in non-smokers’ saliva samples. An essential finding of the research was also that the *Candida* species accounted for the largest percentage of microbes amongst smokers with halitosis [97]. Al-Zyound et al. performed tests showing an increased level of three bacterial genera in smokers: *Streptococcus*, *Prevotella*, and *Veillonella*. Researchers provided evidence that tobacco smoking has a direct effect on the oral microbiota. They also suggested that after smoking cessation, it is possible to return to the standard composition of the oral cavity microbiota [115].

Wu et al. noticed significant changes in the oral microbiota that occurred amongst obese people suffering from malodor. The *Prevotella*, *Granulicatella*, *Peptostreptococcus*, *Solobacterium*, *Catonella*, and *Mogibacterium* were more abundant genera in the obesity group than in healthy persons [116].

Halitosis has often been reported amongst the symptoms related to *Helicobacter pylori* infection and gastroesophageal reflux disease. Anbari et al. made the observations that the incidence of malodor amongst *Helicobacter pylori*-positive patients was 74% [2]. However, Tagerman et al. disagreed about a possible relationship between *Helicobacter pylori* infection and objective halitosis [22].

It is difficult to identify bacteria that promote malodor in children. The most common groups of oral bacteria in children with IOH are *Veillonella* spp., *Prevotella* spp., *Fusobacterium* spp. However, there is no difference in the abundance of these microorganisms in children with IOH and those without [110].

In Table 3, results of studies concerning microbiota associated with IOH are presented. Summarizing the table, the oral bacteria that are most related to IOH are *Actinomyces* spp., *Bacteroides* spp., *Dialister* spp., *Eubacterium* spp., *Fusobacterium* spp., *Leptotrichia* spp., *Peptostreptococcus* spp., *Porphyromonas* spp., *Prevotella* spp., *Selenomonas* spp., *Solobacterium* spp., *Tannerella forsythia*, and *Veillonella* spp.

## 5. Conclusions

The IOH is formed by volatile compounds, among which volatile sulfur compounds (VSCs), such as hydrogen sulfide, dimethyl sulfide, dimethyl disulfide, and methyl mercaptan, are predominant. VSCs are produced mainly by anaerobic bacteria belonging to genera *Actinomyces*, *Bacteroides*, *Dialister*, *Eubacterium*, *Fusobacterium*, *Leptotrichia*, *Peptostreptococcus*, *Porphyromonas*, *Prevotella*, *Selenomonas*, *Solobacterium*, *Tannerella*, and *Veillonella*. A combination of different microbial techniques is recommended to analyze the etiological microflora associated with IOH. Increased knowledge of the microbiota of the oral cavity and especially tongue biofilm is essential for further research to develop new halitosis therapy strategies.

## Figures and Tables

**Table 1:** Volatile compounds present in halitosis [23,30,31,32,33,44,47,48].

Group of Compounds	Compound Name	Chemical Formula	Chemical Structure	Odor Threshold (ppm) [49,50,51,52]	Toxicity in Rats LD_50_ (mg/kg)
Volatile sulfur compounds (VSC)	Hydrogen sulfide	H_2_S	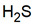	0.00004	15 [53]
	Methyl mercaptan	CH_4_S	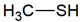	5.1 × 10^−13^	61 (unspecified mammal species) [54]
	Dimethyl sulfide	C_2_H_6_S	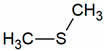	0.00012	3300 [54,55]
	Dimethyl disulfide	C_2_H_6_S_2_	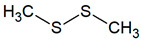	0.00029	190 [54]
	Dimethyl trisulfide	C_2_H_6_S_3_	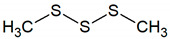	no data	no data
	Allyl methyl sulfide	C_4_H_8_S	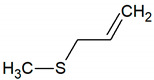	0.00014	no data
Aromatic compounds	Pyridine	C_5_H_5_N	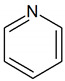	0.01	360–891 [54,55]
	Picoline	C_6_H_7_N	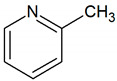	0.0026	200–790 [54,55]
	Indole	C_8_H_7_N	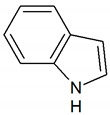	0.0003	1000 [54,55]
	Skatole	C_9_H_9_N	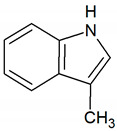	0.0000056	3450 [54,55]
Amines	Ammonia	H_3_N	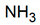	0.043	350 [56]
	Urea	CH_4_N_2_O	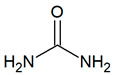	no data	567–8471 [54,55]
	Methylamine	CH_5_N	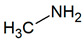	0.00075	100 [54,55]
	Dimethylamine	C_2_H_7_N	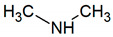	0.00076	698 [54,55,57]
	Trimethylamine	C_3_H_9_N	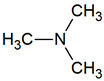	0.00002	500–535 [54,55]
	Putrescine	C_4_H_12_N	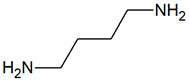	no data	463–2000 [54,55,58]
	Cadaverine	C_5_H_14_N	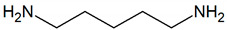	no data	2000 [58]
Short/medium fatty or organic acids	Acetic acid	C_2_H_4_O_2_	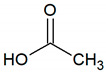	0.0004	3310 [54,55]
	Propionic acid	C_3_H_6_O_2_	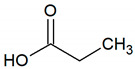	0.00099	2600–3500 [54,55]
	Butyric acid	C_4_H_8_O_2_	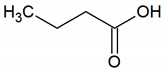	0.001	1500–2000 [54,55]
	Valeric acid	C_5_H_10_O	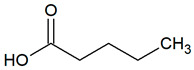	0.000037	2000–4600 [59]
	Isovaleric acid	C_5_H_10_O	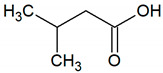	0.000078	2 [54]
Alcohols	Methanol	CH_4_O	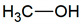	3.05	2131–7529 [54,55]
	Ethanol	C_2_H_6_O	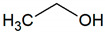	0.09	1440–7060 [54,55]
	Propanol	C_3_H_8_O	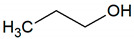	0.031	590–2200 [54,55]
Aliphatic compounds	Cyclopropane	C_3_H_6_	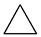	no data	no data
	Cyclobutane	C_4_H_8_	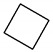	no data	no data
	Pentane	C_5_H_12_	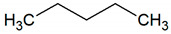	1.29	400–>2000 [54,55]
Aldehydes and ketones	Acetaldehyde	C_2_H_4_O	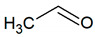	0.0015	640–1930 [54,55]
	Acetone	C_3_H_6_O	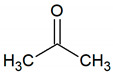	0.4	5500–5800 [54,55,57]
	Acetophenone	C_8_H_8_O	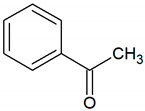	0.00024	815–2650 [54,55]
	Benzophenone	C_13_H_10_O	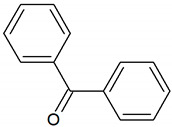	no data	>10,000 [54,55]

**Table 2:** Bacterial producers of volatile sulfur compounds (VSC) [30,96].

Chemical Compound	Bacteria
Hydrogen sulfide from L-cysteine	*Bacteroides intermedius*, *Bacteroides* spp., *Capnocytophaga ochracea*, *Centipeda periodontii*, *Eikenella corrodens*, *Eubacterium brachy*, *E. limosum*, *Eubacterium* spp., *Fusobacterium alocis*, *F. nucleatum*, *F. periodonticum*, *F. sulei*, *Peptostreptococcus anaerobius*, *P. micros*, *P. prevotii*, *Porphyromonas endodontalis*, *Propionibacterium propionicum*, *Selenomonas artemidis*, *S. dianae*, *S. flueggei*, *S. infelix*, *S. noxia*, *S. sputigena*, *Tannerella forsythia*, *Veillonella dispar*, *V. parvula*
Methyl mercaptan from L-methionine	*Bacteroides* spp., *Eubacterium* spp., *F. nucleatum*, *F. periodonticum*, *Porphyromonas endodontalis*
Hydrogen sulfide from serum	*Bacteroides gracilis*, *B. intermedius*, *B. loescheii*, *B. oralis*, *Eubacterium lentum*, *Eubacterium* spp., *F. nucleatum*, *Mitsuokella dentalis*, *Peptostreptococcus magnus*, *P. micros*, *P. prevotii*, *P. propionicum*, *Porphyromonas gingivali*s, *T. forsythia*, *Treponema denticola*, *V. parvula*
Methyl mercaptan from serum	*P. endodontalis*, *P. gingivalis*, *T. denticola*

**Table 3:** Results of studies concerning bacteria associated with intra-oral halitosis (IOH).

Bacteria Related to Intra-Oral Halitosis	Studied Population	Study Method	Reference
*Bacteroides gracilis, B. intermedius, B. loescheii, B. oralis, Capnocytophaga ochracea, Centipeda periodontii, Eikenella corrodens, Eubacterium brachy, E. lentum, E. limosum, Fusobacterium alocis, F. nucleatum, F. periodonticum, F. sulei, Mitsuokella dentalis, Peptostreptococcus anaerobius, P. magnus, P. micros, P. prevotii, Porphyromonas endodontalis, P. gingivalis, Propionibacterium propionicum, Selenomonas artemidis, S. dianae, S. flueggei, S. infelix, S. noxia, S. sputigena, Tannerella forsythia, Treponema denticola, Veillonella dispar, V. parvula*	9 persons	Bacterial culture	[96]
*Fusobacterium* sp., *P. gingivalis, Prevotella intermedia*	16 IOH adults or children	Bacterial culture	[117]
*Campylobacter rectus, F. nucleatum, P. micros, P. gingivalis, P. intermedia, T. forsythia*	40 IOH patients	Anaerobic culture	[118]
*Fusobacterium* sp., *P. gingivalis, P. intermedia, T. forsythia*	20 IOH adults	Anaerobic culture	[119]
*P. gingivalis*, *P. intermedia*, *P. melaninogenica*, *P. nigrescens*, *Streptococcus constellatus*, *T. forsythia*, *T. denticola*, *V. parvula*	10 adult persons	checkerboard DNA-DNA hybridization technique	[120]
*Actinomyces israelii, A. neuii, A. odontolyticus, Aggregatibacter actinomycetemcomitans (serotype a), Atopobium parvulum, Prevotella bivia, P. disiens, P. nigrescens, Pseudomonas aeruginosa, Staphylococcus epidermis, S. constellatus, Streptococcus mitis, T. forsythia, V. parvula*	21 IOH adults	Checkerboard DNA-DNA hybridization	[121]
*F. nucleatum, P. gingivalis, T. forsythia*	30 adults	PCR	[122]
*P. gingivalis, P. intermedia, T. forsythia*	101 IOH adults	PCR	[123]
*P. gingivalis, P. intermedia, P. nigrescens, T. forsythia, T. denticola*	29 IOH patients and 10 healthy adults	Real-time PCR	[124]
*F. nucleatum*, *Solobacterium moorei*, *T. forsythia*	78 adult males	Quantitative real-time PCR	[35]
*A. actinomycetemcomitans, F. nucleatum, P. gingivalis, P. intermedia, T. denticola*	31 IOH patients and 31 healthy adults	16S rDNA-directed PCR	[125]
*Atopobium* sp., *Dialister* sp., *Eubacterium* sp., *Fusobacterium nucleatum, Leptotrichia* sp., *Megasphaera* sp., *Neisseria* sp., *Parvimonas* sp., *Peptococcus* sp., *Peptostreptococcus* sp., *P. gingivalis, P. endodontalis, Prevotella* sp., *Selenomonas* sp., *Solobacterium* sp., *SR1* sp., *Veillonella* sp.	30 IOH patients and 13 healthy persons	PCR and sequencing	[107]
*A. odontolyticus, F. periodonticum, Leptotrichia* sp., *Okadaella gastrococcus, Prevotella melaninogenica, S. moorei, T. forsythia*	6 IOH patients and 6 healthy adults	PCR and sequencing	[112]
phyla Firmicutes and Fusobacteria, genera *Atopobium, Campylobacter, Leptotrichia, Megasphaera, Oribacterium*	26 full dentures patients	PCR and sequencing	[113]
*A. odontolyticus, Atopobium parvulum, Lysobacter*-type species, *Porphyromonas *sp., *P. melaninogenica, P. pallens, P. veroralis, Streptococcus salivarius, S. mitis, S. oralis, V. parvula*	20 IOH patients and 12 healthy adults	PCR and DNA sequencing	[126]
*Eubacterium* sp., *Dialister* sp., *Granulicatella elegans*, *Porphyromonas* sp., *P. intermedia, Staphylococcus warneri, S. moorei*	8 IOH patients and 5 healthy adults	PCR and DNA sequencing	[127]
*Aggregatibacter* sp., *A. segnis*, *Campylobacter* sp., *Capnocytophaga* sp., Clostridiales, *Dialister* sp., *Leptotrichia* sp., *Parvimonas* sp., *Peptostreptococcus* sp., *Peptococcus* sp., *Prevotella* sp., *Selenomonas* sp., SR1, *Tannerella* sp., TM7-3, *Treponema* sp.	16 IOH patients and 10 healthy adults	16S rRNA sequencing	[5]
*Prevotella* sp., *Leptotrichia* sp., *Actinomyces* sp., *Porphyromonas* sp., *Selenomonas* sp., *Selenomonas noxia*, *Capnocytophaga ochracea*	5 IOH children and 5 healthy	16S rRNA sequencing	[128]
*A. parvulum*, *Eubacterium sulci*, *F. periodonticum*, *Dialister* sp., *S. moorei, Streptococcus* sp., TM7-8,	6 IOH patients and 5 healthy adults	16S rRNA sequencing	[129]
*A. odontolyticus, Hemophilus parainfluenzae, Gemella* sp., *Leptotrichia wadei, Prevotella tannerae, Streptococcus* sp.,	29 adults	16S rDNA amplicon sequencing	[130]
*Actinomyces* sp., *Prevotella* sp., *Veillonella* sp.	10 adults	16S rRNA gene sequencing	[131]
*Aggregatibacter* sp., *Anaerovorax* sp., Bacteroidales, *Butyrivibrio* sp., *Dialister* sp., *Eikenella* sp., *Mogibacterium* sp., *Moraxella* sp., *Peptococcus* sp., Peptostreptococcaceae, RF39, *Tannerella* sp., *Treponema* sp., Veillonellaceae	40 IOH adults	16S rRNA sequencing	[132]
*Streptococcus halitosis* sp. nov. strain VT-4	-	16S rRNA sequencing	[133]
